# Morphological and transcriptomic responses/acclimations of erect‐type submerged macrophyte *Hydrilla verticillata* both at low‐light exposure and light recovery phases

**DOI:** 10.1002/ece3.10583

**Published:** 2023-10-06

**Authors:** Qingchun Guo, Yuxuan Gao, Chao Song, Xinhou Zhang, Guoxiang Wang

**Affiliations:** ^1^ School of Environment Nanjing Normal University Nanjing China; ^2^ State Key Laboratory of Vegetation and Environmental Change Institute of Botany, Chinese Academy of Sciences Beijing China

**Keywords:** acclimation, *Hydrilla verticillata*, light compensation point, morphological trait, submerged macrophyte, transcriptome

## Abstract

Light intensity is a determinant for submerged macrophytes. Little is known about their molecular responses to low‐light exposure, despite more informative and responsive than morphological traits. For erect‐type submerged macrophytes, the stem is more crucial relative to the leaf in acclimation to low‐light stress, but receives less attention. We determined morphological and stem transcriptomic responses/acclimations of *Hydrilla verticillata* to extremely and mildly low light (7.2 and 36 μmol photons m^−2^ s^−1^, respectively), that is, EL and ML, with the radiation intensity of 180 μmol photons m^−2^ s^−1^ as the control. Low‐light exposure continued for 9 days, followed by a 7‐day recovery phase (180 μmol photons m^−2^ s^−1^). At the exposure phase, the low‐light treatments, in particular the EL, decreased the relative growth ratio, but induced greater height and longer stem internode distance and epidermal cell. Such responses/acclimations continued into the recovery phase, despite more or less changes in the magnitude. Transcriptome showed that the photosynthetic system was inhibited at the exposure phase, but the macrophyte adjusted hormone synthesis relating to cell division and elongation. Moreover, the EL activated cell stress responses such as DNA repair. Following light recovery, the macrophyte exhibited a strong‐light response, although energy metabolism enhanced. Especially, the EL enriched the pathways relating to anthocyanin synthesis at such phase, indicating an activation of photoprotective mechanism. Our findings suggest that negative influences of low light occur at both low‐light exposure and recovery phases, but submerged macrophytes would acclimate to light environments. Transcriptome can show molecular basis of plant responses/acclimations, including but not limited to morphology. This study establishes a bridge connecting morphological and molecular responses/acclimations.

## Graphical abstract

Negative influences of low light occur both at the low‐light exposure and recovery phases, but submerged macrophytes would acclimate to light environments. The transcriptome can show molecular basis of plant responses/acclimations, including but not limited to the morphology.

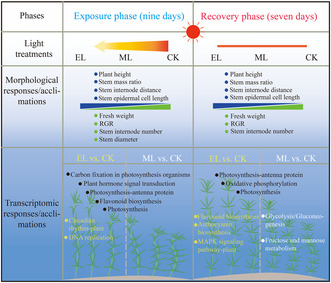

## 1 INTRODUCTION

Submerged macrophytes play an irreplaceable role in maintaining the structure and functions of shallow lake ecosystems (Scheffer et al., 2001), which provide food for heterotrophic organisms, and also serve as shelter for a large number of animals (Paice et al., 2017; Wang et al., 2023). Moreover, the macrophytes can absorb excessive nutrients, inhibit algal growth, stabilize the sediment, and release oxygen (Liu et al., 2020; Paice et al., 2017), and thus help maintain shallow lake in the clear water state (Scheffer et al., 2001). For aquatic ecosystem health, therefore, the growth performance of submerged macrophytes is considered as a main indicator. Light intensity, a critical determinant for plant growth and metabolism, plays a decisive role in controlling the productivity and distribution of submerged macrophytes (Rao et al., 2021). Water level rise, as well as turbidity increase, generally induces low‐light exposure on submerged macrophytes, and such scenario is becoming more frequent due to extreme precipitation events, eutrophication, and reservoir construction (Munoz et al., 2018; Voesenek & Bailey‐Serres, 2015). Numerous studies have shown that low‐light environments generally inhibit the growth of submerged macrophytes (Gao et al., 2021; Yuan et al., 2016), though they have the ability of acclimatized adjustment (Wang et al., 2021; Yang et al., 2022). After elimination of the low‐light exposure, these macrophytes may gradually return to a normal state, or alternatively, continue to maintain a low growth rate, due to the legacy of low‐light exposure (Ibanez et al., 2012). For community stability and ecosystem resistance, whether and how the submerged macrophytes recover from the low‐light damage are remarkable issues.

Morphological traits, such as plant height, leaf number, root:shoot ratios, and cell length, are measurable properties relating to productivity and acclimation to the environment (He et al., 2019). They contain a great deal of information involving many patterns and processes, such as phylogenetic signals, physiological functions, and environmental constraints, at a wide range of scales (Visakorpi et al., 2023). Thus, the morphological plasticity is widely adopted to investigate the vulnerability of macrophytes to environmental change. For instance, submerged macrophytes are found to adjust morphological traits to light shortage, such as decreasing dry matter contents, thinning and elongating stems, and increasing leaf number (Chen et al., 2020). However, the morphological responses/acclimations often delay, and even are undetectable and variable (Chen et al., 2020; van der Plas et al., 2020). By contrast, the gene expression generally responds quickly to external interference and acts as the molecular basis of morphological plasticity (Akman et al., 2016; Whitehead, 2012). Transcriptome analyses can provide insights into gene expression information, and help build a bridge between gene expression and morphological performance. By analyzing the whole transcriptome of *Posidonia oceanica*, Ruocco et al. (2021) found several epigenetic mechanisms, and suggested that the cross‐talk between DNA methylation and the cellular energetic status regulates shoot metabolism at low irradiance levels. Davey et al. (2018) demonstrate that *Zostera muelleri* tends to amplify the expression of genes, relating to photosynthesis, carbohydrate metabolism, and abscisic synthesis, to cope with low‐light exposure. Thus, transcriptome analysis is an effective measure for revealing plant acclimation strategy under unfavorable conditions.

In contrast with the leaf, the stem receives limited attention when exploring how submerged macrophytes acclimate to environmental changes. The truth is, though, the stem also plays a pivotal role in plant acclimation to low‐light environments (Sun et al., 2004). Especially for erect‐type submerged macrophytes, the stem helps the leaf capture light energy by elongating toward the water surface, but also participates directly in primary production (Liu et al., 2021). Therefore, the responses of stem should be taken into account to unravel macrophyte acclimation to low‐light exposure. *Hydrilla verticillata*, a typical erect‐type submerged macrophyte, is widely distributed in freshwater ecosystems in Eurasia. However, relatively little is known about how the stem acclimates to declining light intensity. Here, based on a microcosm experiment, we determined stem transcriptomic responses/acclimations, as well as morphological responses/acclimations, to low‐light exposure, and also concerned the performance after recovery of light conditions. For this study, we hypothesized that (1) *H. verticillate* would cope with low‐light exposure by morphological acclimations; (2) the growth of *H. verticillata* cannot well return to normal after the recovery of light intensity; and (3) transcriptome can provide detailed information on responses/acclimations, including but not limited to the molecular basis of morphological performances.

## 2 MATERIALS AND METHODS

### 2.1 Experimental design

*Hydrilla verticillata* shoots were collected from Lake Taihu (31°20′47″ N, 120°28′41″ E), a shallow lake with a water area of 2338 km^2^ and an average water depth of 1.9 m. The top 8‐cm segments were cut from healthy individuals, weighed, and used as the asexual propagules in this experiment. After rinsed with distilled water, the propagules were planted in plastic bottles (10 cm diameter, 40 cm height, and eight propagules in each bottle) containing 2 L distilled water and 200 g sediment, with total organic carbon 12.8 mg g^−1^, total nitrogen 2.73 mg g^−1^, and total phosphorus 0.730 mg g^−1^. These propagules in bottles were pre‐incubated at 27°C for 15 days in three growth chambers, with the light–dark regime of 12 h:12 h and the radiation intensity of 180 μmol photons m^−2^ s^−1^. Following the pre‐incubation, based on the light compensation point of *H. verticillata* (i.e., 15.8 μmol photons m^−2^ s^−1^, Su et al., 2004), the light intensity of two chambers were, respectively, lowered to 7.2 and 36 μmol photons m^−2^ s^−1^. These two chambers represented an extremely low‐light environment (EL) and a mildly low‐light environment (ML), respectively, with another chamber still maintained the light intensity at 180 μmol photons m^−2^ s^−1^ (CK). According to the mean duration of flood in Lake Taihu in the past 30 years, the low‐light exposure continued for 9 days in this study. Subsequently, the light intensity of both EL and ML treatments returned to 180 μmol photons m^−2^ s^−1^, and the recovery phase continued for 7 days. For this experiment, there were six bottles evenly placed in each growth chamber, and transposition was carried out every day to avoid unequal light radiation. Moreover, distilled water was supplemented as needed to maintain a constant water level throughout the experiment.

### 2.2 Determination of morphological traits

At the end of both low‐light exposure and light recovery phases, we chose three replicated plastic bottles from each growth chamber and determined morphological parameters of *H. verticillata*. Two healthy individuals from each plastic bottle were harvested, washed, and dried with filter paper to determine the total biomass, the number of stem internode, and the average distance of stem internode. In addition, plant height and stem diameter (at 5 cm down the tip) were measured with a ruler (0.1 cm precision) and a micrometer (0.02 mm precision), respectively. After the measurement, the *H. verticillata* individuals in each plastic bottle were separated into leaves, stems, and roots, oven‐dried at 80°C to a constant weight, and then weighed to obtain the dry weight. The stem mass ratio was defined as the ratio of stem dry weight to total dry weight. Relative growth rate (RGR) was calculated as

$$\mathrm{RGR}=\frac{lnW_{2}-\ln W_{1}}{\Delta T}$$

where *W*_1_ is the initial fresh weight (mg), *W*_2_ is the final fresh weight (mg), and ∆*T* is the time (day). In addition, a scan with the electron microscope (JSM‐5610LV, Thermo Fisher Scientific) was used to determine stem epidermal cell length (at least 15 cells per sample) at 5 cm down the tip.

### 2.3 RNA extraction and sequencing

In addition to the morphological parameters, the apical 5‐cm stem of *H. verticillat*a was clipped, shock‐frozen in liquid nitrogen, stored at −80°C, and used for transcriptome analysis. Total RNA was extracted from the stem samples with RNA 6000 Nano kit (Agilent). RNA concentration and purity were determined by UV spectrophotometry, and the integrity was assessed by an Agilent 2100 Bioanalyzer (Agilent). The total amount of RNA required for a single library construction is ≥1 μg, the concentration is ≥35 ng/μL, OD_260_/_280_ is ≥1.8, and OD_260_/_230_ is ≥1.0. In this experiment, all RIN (RNA Integrity) numbers were above eight, indicating that high‐quality material was used for sequencing. Fifteen indexed cDNA libraries, that is, (three light treatments at the end of the low‐light exposure phase + two low‐light treatments at the end of light recovery phase) × three replicates, were constructed and sequenced with an Illumina Novaseq 6000 platform (Paired‐ends 2 × 150 cycles).

### 2.4 Transcriptome assemble and functional annotation

Samples were collected at the end of both low‐light exposure and light recovery phases, and transcripts were spliced after each sampling. For this study, the cDNA libraries of CK treatment constructed at the end of the low‐light exposure phase were as the control both at the low‐light exposure and recovery phases. Sequence raw reads were checked for data quality using FastQC. Cutadapt was used to remove sequences with adapters at the 3′ end and reads with an average quality score below Q20. We used Trinity with default parameters to assemble clean reads into a transcriptome, as there is no available reference genome for *H. verticillata*. The longest transcript was selected and used for subsequent analysis. Benchmarking Universal Single‐copy Orthologs (BUSCOs) was used to complete the integrity of the assembled transcriptome.

Assembled transcripts were annotated through sequence similarity search against UniProt databases with Blast+ software (e‐value cut‐off 1e−5). Subsequently, results were loaded on Blast2GO v.6 to retrieve Gene Ontology terms (e‐value cut‐off 1e−6). KO assignment and Pathway mapping were performed with KAAS (https://www.genome.jp/tools/kaas/).

### 2.5 Gene expression and functional enrichment analyses

RSEM with default parameters was used to estimate the gene expression level by calculating the read count of each transcript. We used “edge R” package to perform differential analysis of high‐abundance genes (≥1 read/count per million). Transcripts were considered differentially expressed when FDR < 0.05 and logFC > ±1.

The differentially expressed genes (DEGs) were further analyzed by KEGG enrichment analysis with the online platform (https://www.omicshare.com/tools). The ggord package based on R was used for Principal Co‐ordinates Aanalysis (PCoA) to reflect the similarity between samples. Venn diagram (http://www.ehbio.com/test/venn/#/) was used to show the distribution of the number of DEGs that were unique and shared by ML versus CK and EL versus CK at the low‐light exposure and light recovery phases. The heatmap showed the expression levels of genes in KEGG pathways at the low‐light exposure and light recovery phases. Transcriptomics data used in this study are publicly available from NCBI (BioProject ID PRJNA977960).

### 2.6 Statistical analysis

All statistical analyses were performed with R (v. 4.2.3, R Core Team, 2023) with an accepted significance level of *p* < .05. The normality of all data was tested with the Shapiro–Wilk test before statistical analysis. Repeated measures analysis of variance (ANOVA) was used to assess the effects of light treatment, incubation phase, and their interactions on morphological traits. Following one‐way ANOVA, Tukey's multiple comparison test was used to detect the differences in plant height, the number of stem internode, the average distance of stem internode, stem diameter, stem mass ratio, fresh weight, and RGR among light treatments at the same phase, that is, the low‐light exposure phase or the light recovery phase.

## 3 RESULTS

### 3.1 Morphological traits

**FIGURE 1:**
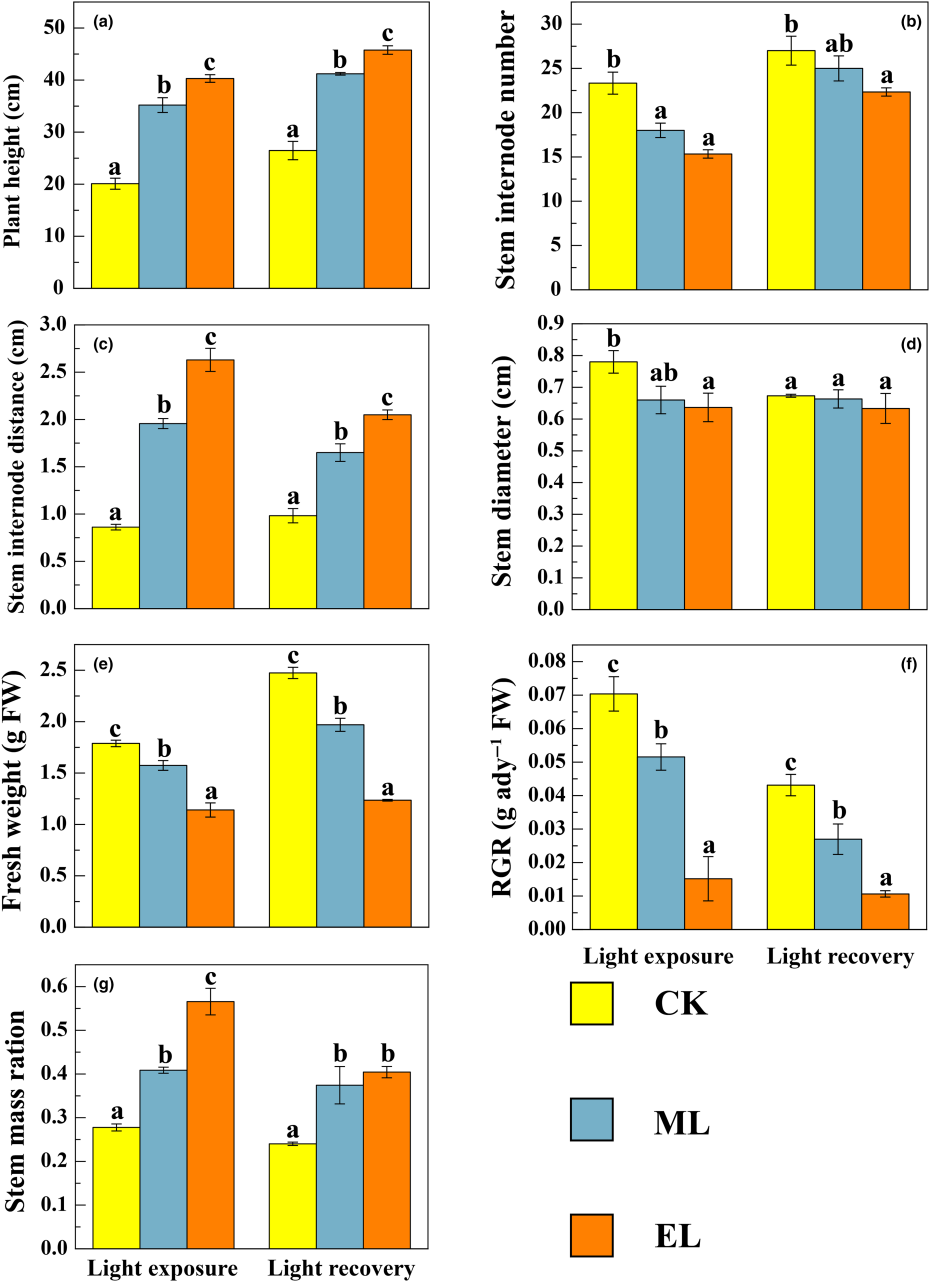
Responses of plant height (a), internodes number (b), stem internodes distance (c), stem diameter (d), fresh weight (e), RGR (f), stem mass ratio (g) to light intensity at the low‐light exposure and light recovery phases. Data are means and error bars are SD (*n* = 3). Different lowercase letters indicate significant differences between light irradiance treatments at the same phase (*p* < .05).

**FIGURE 2:**
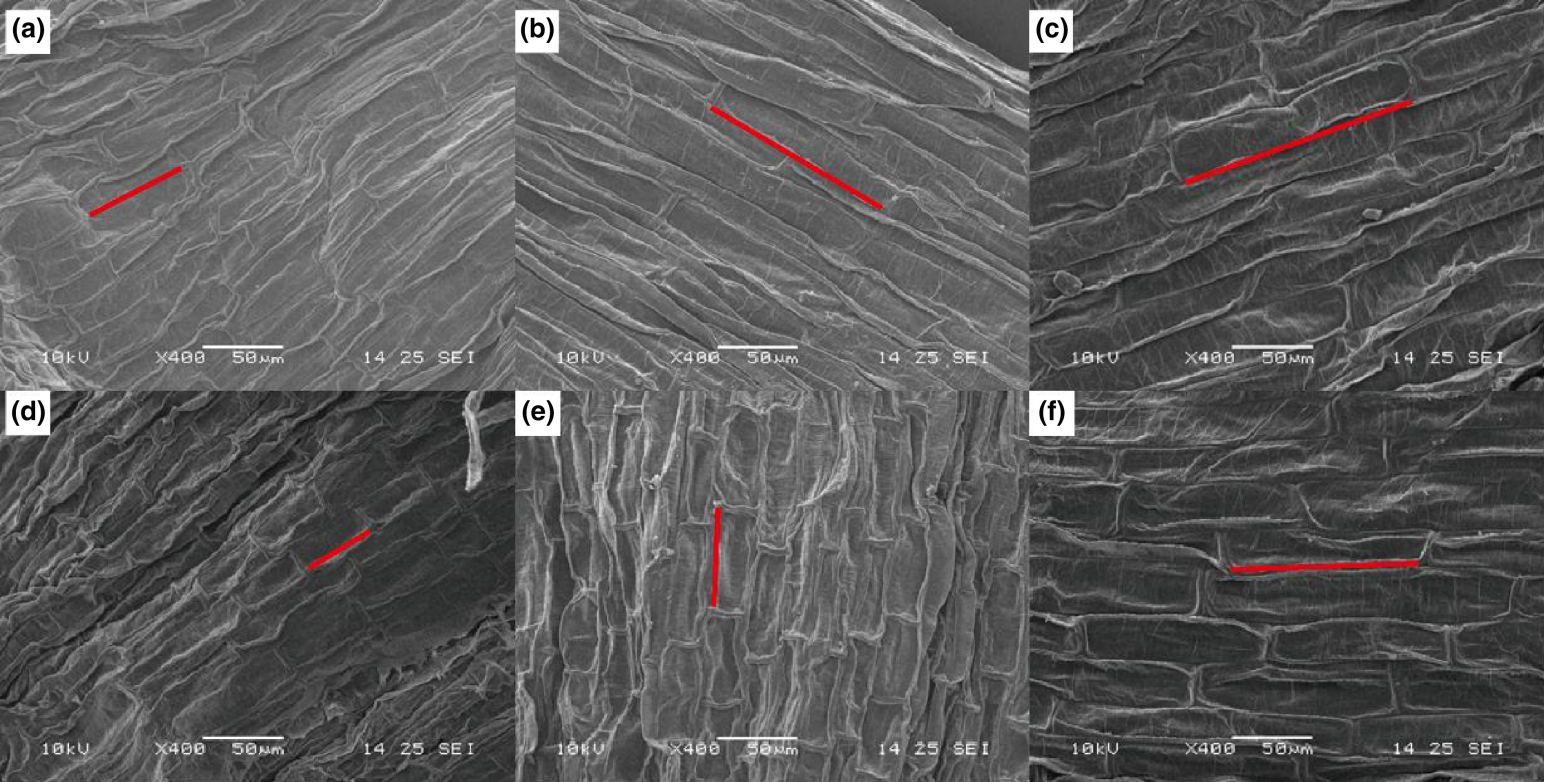
SEM micrograph showing the stem epidermal cell length of *Hydrilla verticillate* at the low‐light exposure (a: CK; b: ML; c: EL) and light recovery phases (d: CK; e: ML; f: EL).

Light irradiance intensity generally exerted significant effects on morphological traits, and the effects on plant height and stem internode number did not vary significantly between the low‐light exposure and the recovery phases (Table 1; Figure 1). At the end of low‐light exposure phase, the decreased light irradiance, especially the EL treatment, induced significant increases in plant height, stem internode distance, stem mass ratio, as well as significant decreases in internode number, fresh weight, stem diameter, and RGR (Figure 1). Moreover, the length of stem epidermal cells obviously increased with the decrease of light intensity (Figure 2a–c). It is worth noting that such influences from low light continued into the light recovery phase, although the magnitudes had more or less changes (Figures 1 and 2d,e).

**TABLE 1:** Results (*F‐*values) of repeated measures ANOVA indicating effects of incubation phase, light treatment, and their interaction on morphological traits.

	df	Fresh weight	RGR	Plant height	Stem internode number	Stem internode distance	Stem diameter	Stem mass ratio
Within‐subject effect								
Incubation phase	1	179***	36.3*	57.7***	128***	30.2**	14.2**	25.7**
Incubation phase × Light treatment	2	33.8**	5.28*	0.111^ns^	4.55^ns^	19.3**	14.3 **	7.45*
Error	6							
Between‐subject effect								
Light treatment	2	371***	195***	638***	24.8**	383***	3.59^ns^	166***
Error	6							

***p < .001, **p < .01, *p < .05, ^ns^ *p* > .05.

### 3.2 Transcript assembly and annotation results

**FIGURE A1:**
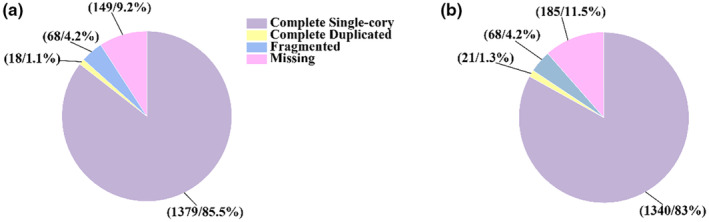
Results of completeness assessment of the *Hydrilla verticillata* transcriptome based on Benchmarking Universal Single‐Copy Orthologs (BUSCOs). (a) Low‐light exposure phase. (b) Light recovery phase.

In this project, a total of 65 million clean reads were produced, with 3.7~4.8 million per sample (Table A1 in Appendix). At the end of the low‐light exposure phase, there are 101,363 unigenes obtained, with a mean length of 876.71 bp and an N50 value of 1268 bp (Table A2 in Appendix). The functional annotation analysis showed that 41,027 (40.48%) and 18,303 (18.06%) unigenes significantly matched NR and KEGG databases, respectively (Table A3 in Appendix). There was a high proportion (86.5%) of complete BUSCOs present, indicating the transcriptome was relatively complete (Figure A1 in Appendix).

At the end of light recovery phase, 117,484 unigenes were observed, with a mean length of 826.74 bp and an N50 value of 1121 bp (Table A2 in Appendix). There were 49,745 (42.34%) and 25,434 (21.65%) unigenes significantly matching NR and KEGG databases, respectively (Table A3 in Appendix). Moreover, the proportion of complete BUSCOs was up to 84.3% (Figure A1 in Appendix).

### 3.3 DEGs analysis

**FIGURE 3:**
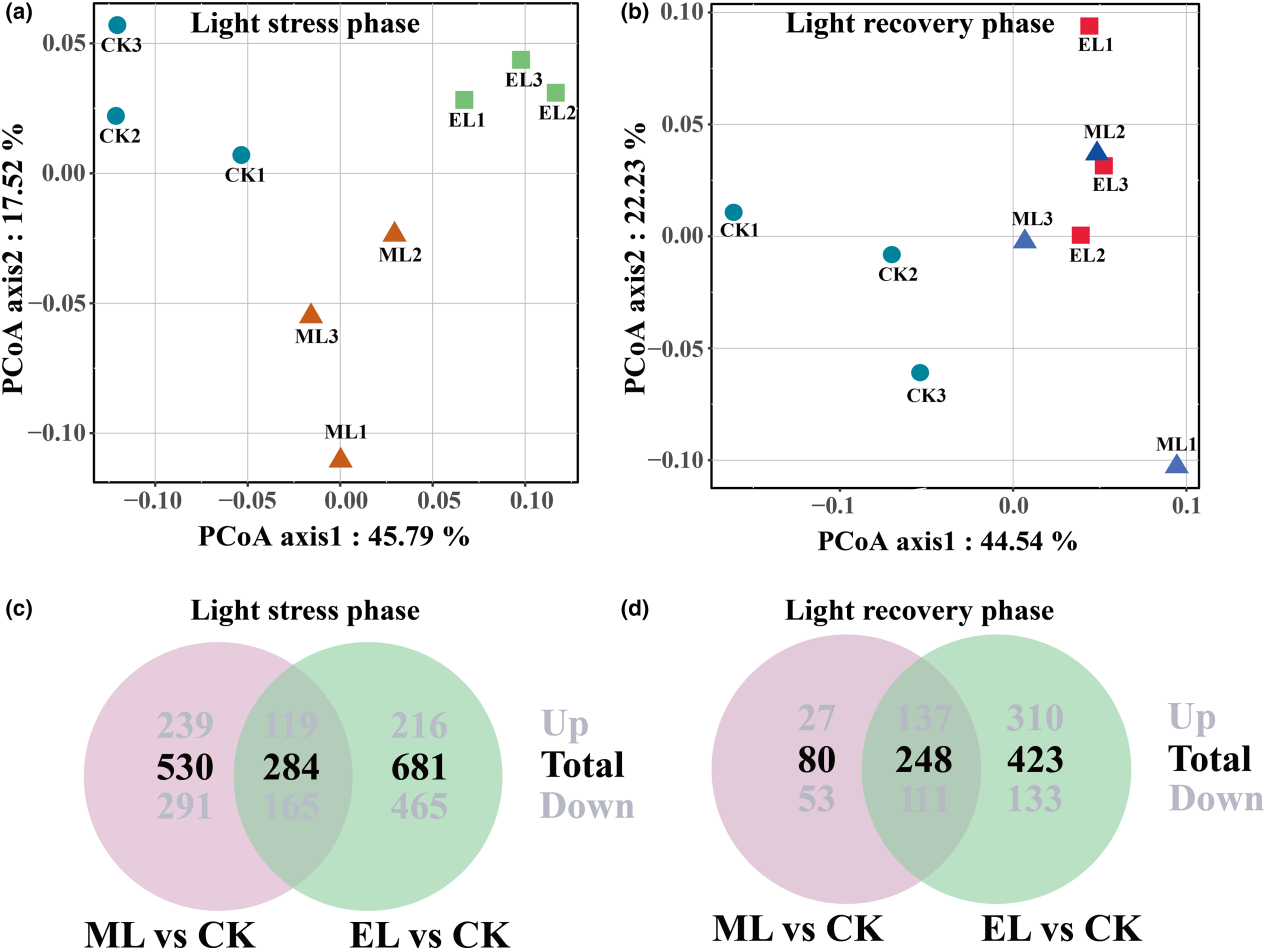
Analysis of gene expression in experimental *Hydrilla verticillatac* plants. PCoA was performed with the normalized expression values of plant genes at the low‐light exposure (a) and light recovery phases (b). Venn diagram depicting shared and unique DEGs in different light treatments at the low‐light exposure (c) and light recovery (d) phases. Bold values denote the DEG number, while gray values above and below the bold value indicate up‐regulated and down‐regulated DEGs.

In contrast with the ML treatment, the EL treatment induced a larger number of DEGs both at the low‐light exposure and light recovery phases (Figure 3).

Nine days into the low‐light exposure phase, a total of 814 DEGs comprising 456 (56.0%) and 338 (44.0%) down‐ and up‐regulated genes, respectively, were detected in ML versus CK (Figure 3c). For EL vs. CK, there were 965 genes differentially expressed, with 630 (65.3%) down‐regulated and 335 (34.7%) up‐regulated genes (Figure 3c). In the up‐regulated DEGs, 119 (17.2%) genes were shared between the two low‐light treatments (Figure 3c). In the down‐regulated DEGs, 165 (15.2%) genes were shared by the two low‐light treatments (Figure 3c). The PCoA showed that PC1 and PC2 axis, respectively, contributed 45.79% and 17.52% of the total variance. The PC1 axis mainly distinguishes the light treatments, while the PC2 axis distinguishes the replicates belonging to the same treatment (Figure 3a).

Seven days into the light recovery phase, there were 164 (50.0%) and 310 (57.0%) up‐regulated genes in ML and EL treatments, respectively, while the number of down‐regulated genes were, respectively, 164 (50.0%) and 244 (43.0%) (Figure 3d). In the DEGs, there were 137 (28.9%) up‐regulated and 111 (27.2%) down‐regulated genes shared by two low‐light treatments (Figure 3d). The PCoA showed that the first axis (44.54% of total variance) mainly separated low‐light treatments from CK, while the difference in the second axis (22.23% of total variance) was caused by the intra‐group differences in the samples (Figure 3b).

### 3.4 KEGG pathway analysis of DEGs

**FIGURE 4:**
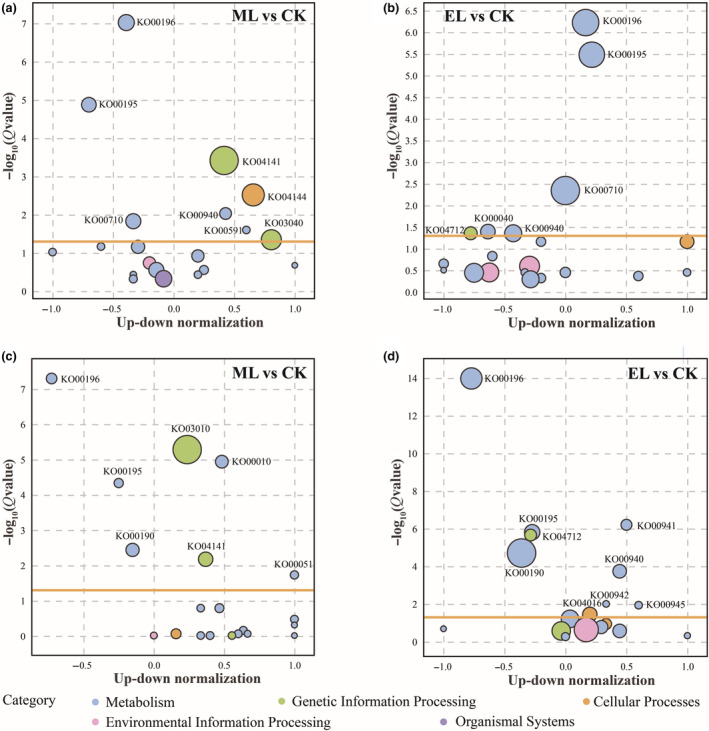
KEGG pathway analysis of DEGs at the low‐light exposure (a, b) and light recovery phases (c, d). The pathways with *Q* value (FDR) < 0.05 were regraded to significantly change. The yellow line represents *Q* value (FDR) = 0.05. The bubble size represents the number of target genes enriched in the current pathway.

**FIGURE 5:**
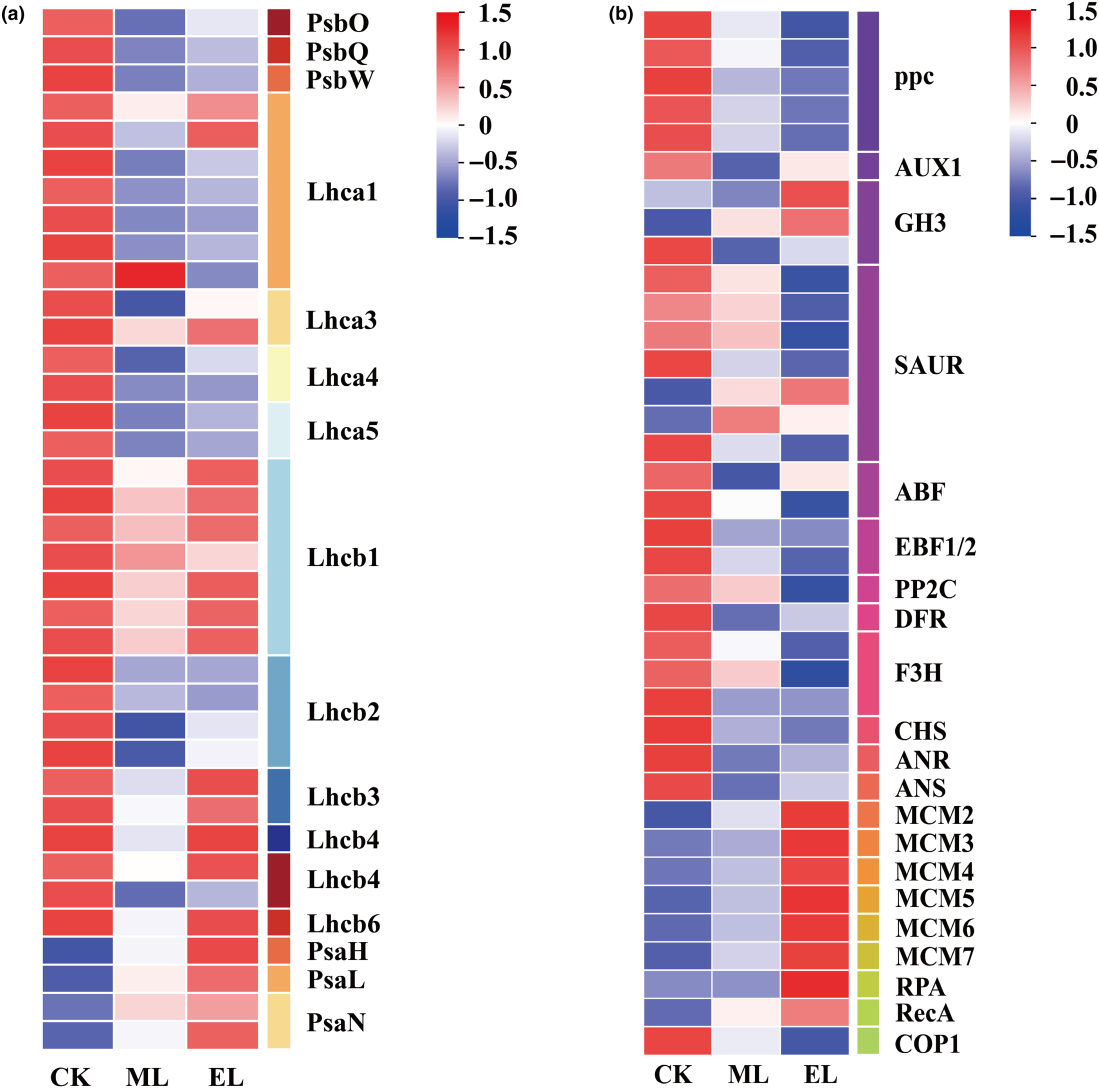
The expression of genes encoding some proteins at the low‐light exposure phase.

At the end of low‐light exposure phase, 814 DEGs were significantly enriched (FDR < 0.05) into eight pathways in the ML treatment, most of which relate to metabolism, such as photosynthesis, photosynthesis‐antenna proteins, and carbon fixation in photosynthetic organisms. In addition, plant hormone signaling transduction (FDR = 0.094) and flavonoid biosynthesis (FDR = 0.18) were also enriched (Figure 4a; Table A4 in Appendix). A similar pattern was also found in the EL treatment, with six pathways significantly affected, including the pathways regulating energy metabolism, carbohydrate metabolism environmental acclimation (circadian rhythm‐plant), and biosynthesis of other secondary metabolites. The DNA repair was enriched (FDR = 0.068) in the EL treatment (Figure 4b; Table A4 in Appendix). In terms of the genes involving in photosynthesis, the genes encoding PsbO and PsbQ proteins were down‐regulated in both low‐light treatments (Figure 5a). However, the genes encoding LHCII were down‐regulated and up‐regulated, respectively, in the ML and EL treatments (Figure 5a). For the genes directly involving plant hormone signal transduction, there were nine and 11 down‐regulated ones in the ML and EL treatments, respectively, and six ones were uniquely up‐regulated in the EL treatment (Figure 5a,b; Table A4 in Appendix). Both low‐light treatments resulted in down‐regulation of genes relating to important enzymes in flavonoid synthesis (anthocyanin synthase (ANS), dihydroflavonol reductase (DFR), chalcone synthase (CHS), anthidin reductase (ANR); Figure 4b; Table A4 in Appendix). In addition, all genes identified involving in DNA repair were up‐regulated in the EL treatment, including six DNA replication licensing factor MCM (2–7) genes (Figure 5b; Table A4 in Appendix).

**FIGURE 6:**
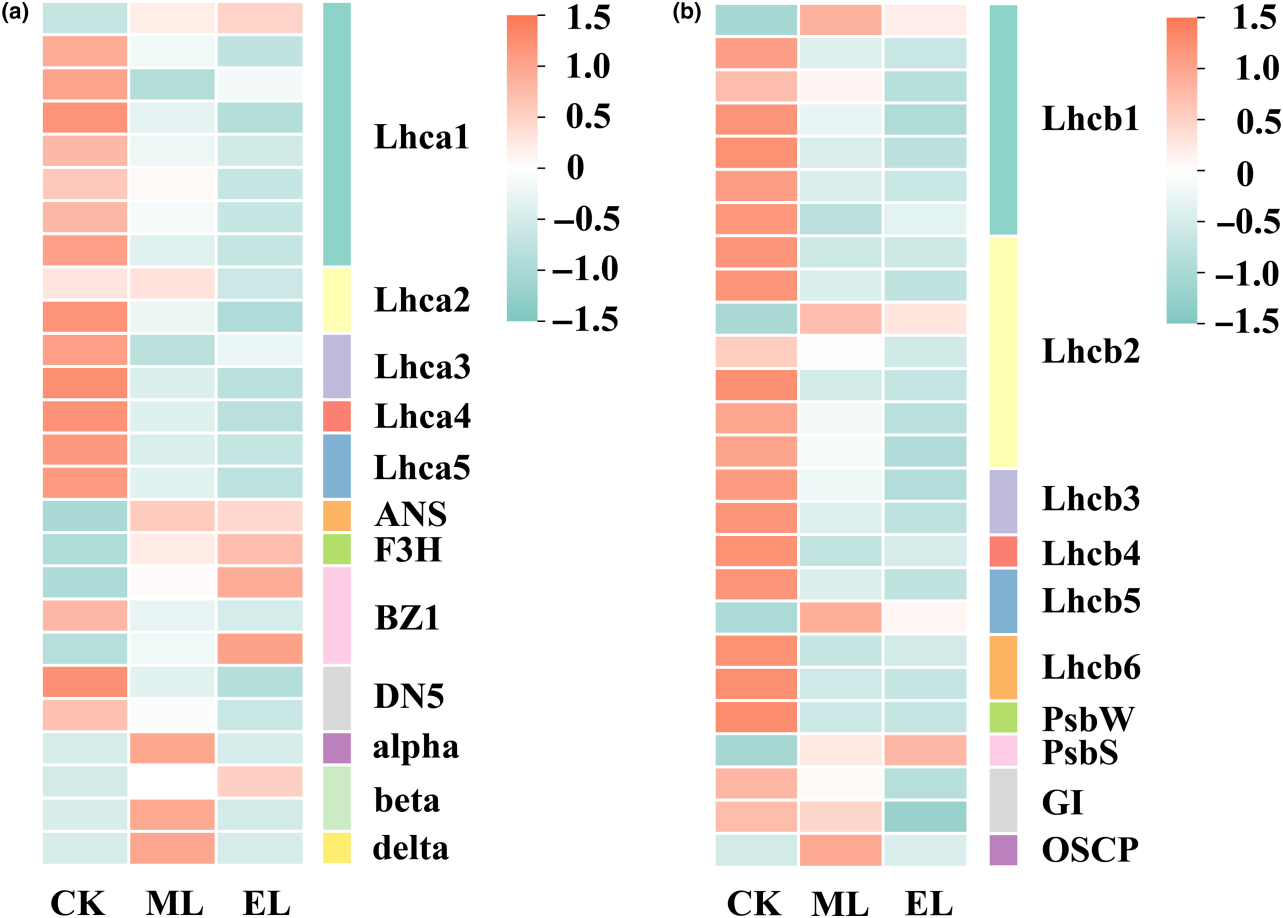
The expression of genes encoding some proteins at the light recovery phase.

At the end of the light recovery phase, 328 DEGs were significantly enriched into seven KEGG pathways in the ML treatment, including photosynthesis‐antenna proteins, flavonoid biosynthesis, circadian rhythm‐plant, anthocyanin biosynthesis, and MAPK signaling pathway‐plant (Figure 4c; Table A5 in Appendix). In the EL treatment, 554 genes were enriched into 9 KEGG pathways. The Glycolysis/Gluconeogenesis was marginally significantly enriched (FDR = 0.060; Figure 4d; Table A5 in Appendix). Twenty‐two and 16 genes involving in photosynthesis were identified as differentially expressed in the EL and ML treatment, respectively, including *psbW* and *psbS*. In photosynthetic‐antenna proteins, most of the differential genes encoding LHCII were down‐regulated in two low‐light treatments, including *Lhcb1*, *Lhcb2*, *Lhcb3*, *Lhcb4*, *Lhcb5*, and *Lhcb6* (Figure 6b; Table A5 in Appendix). In oxidative phosphorylation, 14 of 33 DEGs showed up‐regulation in the ML treatment group, including one gene encoding inorganic pyrophosphatase and one gene encoding F‐type H+‐transporting ATPase subunit alpha. In the EL treatment, 16 of 50 DEGs were upregulated, including one gene encoding F‐type H+‐transporting ATPase subunit alpha, two genes encoding F‐type H+‐transporting ATPase subunit beta, and one gene encoding F‐type H+‐transporting ATPase subunit delta (Figure 6a; Table A5 in Appendix). In addition, we found that genes relating to anthocyanin synthesis were significantly down‐regulated in the EL treatment, including *BZ1*, *ANS*, and *F3H* (Figure 6a).

## 4 DISCUSSION

**FIGURE 7:**
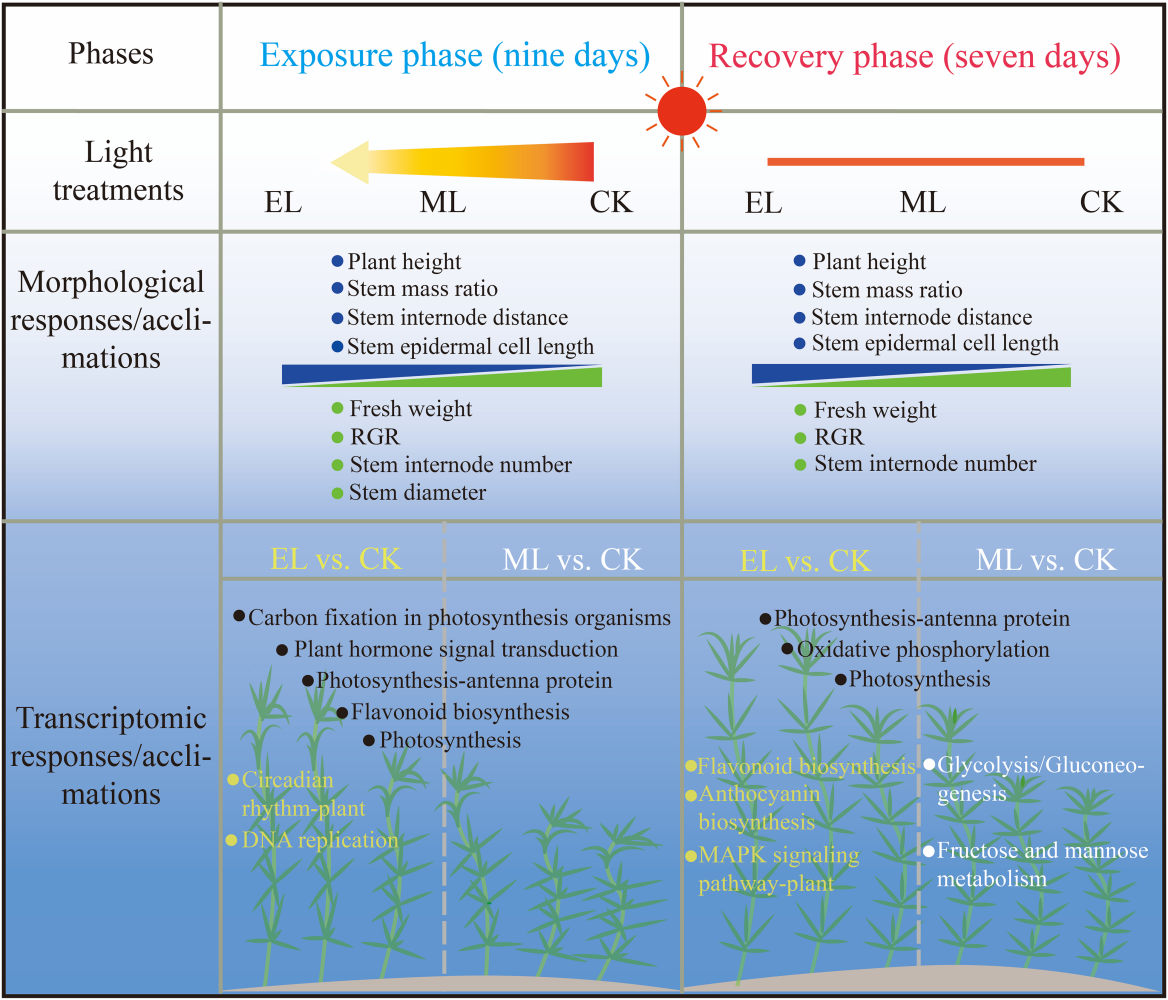
Diagram showing the multi‐level responses/acclimations of *Hydrilla verticillata* to light treatments.

Underwater light intensity is a critical determinant for the survival and growth of submerged macrophytes. Based on an incubation experiment, we investigated morphological and molecular responses/acclimations of *H. verticillata* to low‐light exposure and also paid attention to the performance after elimination of low‐light exposure. We found the morphological traits and the transcriptome expression generally varied with light treatments at both the exposure and the recovery phases (Figure 7). Moreover, the transcriptomic information not only provided the molecular basis of plant morphological traits, but also included unobservable response mechanisms (Figure 7). To the best of our knowledge, this is the first attempt to explore the molecular mechanism behind how submerged macrophytes acclimate the stem for low‐light environments.

### 4.1 Morphological responses/acclimations to low‐light environments

In this study, the low‐light exposure, especially when the light intensity was below the light compensation point, substantially inhibited the growth of *H. verticillata*, just as shown by the low biomass and RGR. This is consistent with the results of many previous studies regarding the effects of light intensity on submerged macrophytes (Chen et al., 2020; Yuan et al., 2016). For example, Yang et al. (2022) found that the biomass of *Vallisneria natans* decreased with the decline of light intensity, and more resources were devoted to clonal reproduction and population stability than to primary production. The decreased photosynthetic products under low‐light conditions can take account for such a phenomenon (He et al., 2019). In addition, plants often synthesize more secondary substances to acclimate to harsh environments, thus further decreasing the matter and energy allocations to growth. Nevertheless, we also found *H. verticillata* positively coped with the low‐light environment in this study. Specifically, *H. verticillata* in low‐light treatments had higher shoots, longer internode distance, thinner stem, longer stem epidemic cell length, and greater stem mass ratio, which could help capture sunlight, thus alleviating low‐light stress (Fu et al., 2012; Strand & Weisner, 2001). Such findings are consistent with our first hypothesis, in which *H. verticillata* is expected to cope with low‐light exposure by morphological acclimations. Similar results have been found in previous studies on canopy‐type submerged macrophytes (e.g., Chen et al., 2020; Fu et al., 2012). For example, Wang et al. (2021) found *Potamogeton crispus* had high plant height, large leaf area, and long leaf length, as well as thin stem in low‐light environments. With the so‐called “light foraging” strategy, the submerged macrophytes can get amounts of light energy at minimal cost under low‐light conditions. When the photosynthetic function of stem is taken into account, such morphological acclimation is becoming more important for the erect‐type submerged macrophytes.

After 7 days of light recovery, the growth of *H. verticillata* did not well return to normal, which is consistent with our second hypothesis. In general, the morphological difference between low‐light treatments and the control obviously remained. For example, there was no significant change in terms of RGR in the EL treatment, which was even lower than that at the low‐light exposure phase in the ML treatment. Presumably, the reason behind such insensitivity may lie in that the plants have no enough energy to acclimate their morphology in time to light recovery, due to extremely low energy storage at the low‐light exposure phase.

Collectively, our findings indicate that low‐light exposure inhibits the growth of erect‐type submerged macrophytes, especially when the light intensity is lower than the light compensation point, in spite of morphological acclimation. Moreover, such inhibition effects can continue into the light recovery phase.

### 4.2 Transcriptomic responses/acclimations to light irradiance

#### 4.2.1 Transcriptomic responses/acclimations at the low right exposure phase

In this study, low‐light exposure significantly affected the photosynthetic capacity of *H. verticillata*. Specifically, as essential proteins for the optimal oxidation activity of photosystem II, the reduced synthesis of PsbO and PsbQ in low‐light treatments indicates an inhibition of photosystem II and thus a decrease in photosynthetic capacity (Wei et al., 2016). Moreover, the expression of the gene encoding ppc protein was significantly down‐regulated in low‐light treatments. As ppc enzymes play an important role in nocturnal CO_2_ assimilation and daytime CO_2_ absorption cycles (Boxall et al., 2020), the present results mean that low light interferes with carbon fixation and energy absorption. Therefore, these findings provided the molecular basis for the inhibition effect of low light on plant growth performance.

Phytohormones control plant growth by regulating gene expression, which is particularly important when plants cope with unfavorable light environments (Curaba et al., 2014). In this study, the low light affected the synthesis of several proteins, such as EBF1/2, ABF, and GH3, in the plant hormone signaling pathway. As EBF1/2 protein is a key component of the ethylene synthesis pathway, the synthesis reduction of which is beneficial to ethylene accumulation in plants, thus promoting elongation of stem internode for submerged macrophytes (Kendrick & Chang, 2008; Kuroh et al., 2018). Abscisic acid is an important plant hormone relating to various aspects of plant development, such as inhibition of cell division and growth. In the synthesis of abscisic acid, the ABF protein acts as a key transcription regulation factor. Therefore, the down‐regulated expression of the transcript encoding the ABF protein is beneficial to the improvement of *H. verticillata* stem growth under low‐light conditions. It is also noteworthy that the transcripts encoding a protein associated with auxin synthesis (GH3) were overexpressed in the EL treatment, which implies oversynthesis of auxin. The auxin is considered to promote the elongation of plant cells, but also plays a regulatory role in the distribution of carbohydrates and energy (Alpert et al., 2002; Javid et al., 2011). Under low‐light conditions, the increase of auxin synthesis can help the plant concentrate energy and carbohydrates into the tip meristem, thus promoting stem elongation. These findings above indicate hormone adjustment in response to low‐light exposure, and further provide a molecular basis for growth performance.

In addition to phytohormones, the secondary metabolites also play a critical role for plants in coping with environmental stress (Erb & Kliebenstein, 2020; Jan et al., 2021). As a common secondary metabolite, flavonoids have multiple functions in physiological metabolism, such as resisting environmental stress and self‐protection (Harborne & Williams, 2000; Nakabayashi & Saito, 2015). In the flavonoid biosynthesis pathway, CHS, DFR, ANS, and ANR are the major intermediate enzymes (Tan et al., 2013). The expression levels of synthetic transcripts encoding these enzymes were down‐regulated under both low‐light treatments, implying that the low‐light exposure reduces the synthesis of flavonoids, and thus induces a decline in defense against environmental disturbance. Consistent with our study, previous studies regarding terrestrial plants also found shade treatment reduced the synthesis of flavonoids (Ye et al., 2021). In addition, the pathways relating to environmental acclimation, such as circadian rhythm‐plant, were uniquely inhibited in the EL treatment. The response of flavonoid synthesis suggests that the environmental adaptability of *H. verticillata* would greatly decrease when the light intensity is lower than the light compensation point. These findings suggest that low‐light exposure would decrease the defense capability of submerged macrophytes, and therefore the plant population would suffer worse impacts when other environmental disturbances happen simultaneously with the low‐light exposure.

In the EL treatment, we enriched certain pathways relating to cellular stress response, such as DNA repair. In the DNA repair, all transcripts associated with the small chromosome maintenance complex (MCM2‐7) were significantly up‐regulated in the EL treatment. Considering that MCM takes part in DNA replication by controlling cell mitosis at the S‐phase (Chang et al., 2007; Shultz et al., 2009), such results indicate a strengthening of DNA respiration under extremely low‐light conditions. These results indicate that the submerged macrophytes would cope with low‐light environments by active cellular stress response, which cannot be found in studies only involving morphological traits. Collectively, these results above support our third hypothesis that transcriptome can provide detailed information on responses/acclimations, including but not limited to the molecular basis of morphological performances.

#### 4.2.2 Transcriptomic responses/acclimations at the light recovery phase

Oxidative phosphorylation is a coupling reaction, in which the energy released during the oxidation of substances supplies ADP and inorganic phosphoric acid to synthesize ATP (Cogliati et al., 2016; Fuchs et al., 2020). Specifically, the expression of the gene encoding inorganic pyrophosphatase was up‐regulated in ML treatment, indicating an over‐synthesis of ATP (Chu et al., 2020). In addition, the pathways associated with carbohydrate metabolism were significantly enriched in ML treatment, such as glycolysis/gluconeogenesis and fructose and mannose metabolism. As one main metabolic pathway of glucose, glycolysis can provide ATP for life activities (Nie et al., 2020). Given that glucose‐6‐phosphate isomerase (GPI) controls the second step of glycolysis, that is, catalyzing the reversible isomerization of glucose‐6‐phosphate and fructose‐6‐phosphate (Haller et al., 2011; Lin et al., 2009), the up‐regulated expression of the gene encoding GPI implies an enhanced glycolysis. Therefore, these results suggest that the recovery of light intensity would boost energy metabolism, and thus contributing to plant growth and recovery.

Despite a potential promotion on plant growth, the shift from low light to normal light exerted substantial disruptions on the photosynthetic system. Non‐photochemical quenching is an important self‐protective mechanism for plants exposed to strong light, where PsbS protein is considered to be the key (Ruban, 2016). The gene encoding PsbS protein was significantly up‐regulated in both low‐light treatments, indicating that the shift of light intensity causes damage to the photosynthetic system of *H. verticillata* stem. As a core component of PSII, moreover, the reduction of pabW protein synthesis indicates reduced stability of PSII and inhibited photosynthesis (Bishop et al., 2003; Shi et al., 2000). Similarly, Hussner et al. (2010) also found the photosynthetic system of submerged macrophytes growing in a low‐light environment was inhibited when they were transferred to a strong‐light environment. Moreover, the transcriptomic information also indicates a self‐protection of *H. verticillata* when it suffers sharp changes in light environments. For instance, the coding genes of antenna proteins, including Lhca1, 2, 3, and 4, as well as Lhcb1, 2, 3, 4, 5, and 6, were down‐regulated, suggesting that the *H. verticillata* acclimates to increased light intensity by reducing light energy transfer (Teramoto et al., 2002). In summary, the submerged macrophytes would be subjected to light damage when the light environment recoveries from a low‐light state, and make acclimatized adjustments to such environment transformation, which may be the reason behind the poor morphological performance of *H. verticillata* at the light recovery phase. Such founding confirms our second hypothesis at the molecular level, that is, *H. verticillata* cannot well return to normal after the recovery of light intensity.

In addition, we also found several unique pathways in the EL treatment. The pathways relating to anthocyanin synthesis, such as flavonoid biosynthesis and anthocyanin biosynthesis, were activated, and the expression of three key protein‐coding genes, including *BZ1*, *ANS*, and *F3H*, were significantly up‐regulated. Considering that anthocyanins are key pigments protecting plant cells against photodamage (Huang et al., 2019), the pathways and genes mentioned above indicate an activation of photo‐protective mechanisms of submerged macrophytes when the light intensity returns to normal level. In addition, the MAPK signaling pathway plant was also significantly enriched. In a previous study (Mizoguchi et al., 1997), the MAPK cascades have been found to take part in higher plants acclimation to environmental stresses. These unique pathways indicate that the submerged macrophytes subjected to extremely low‐light exposure would invest in the photo‐protection when the light conditions returned to normal, presumably which is a significant cause of why these plants had limited increase in the fresh weight at the light recovery phase.

## 5 CONCLUSIONS

In this study, we determined morphological and stem molecular response/acclimation of *H. verticillata* to low‐light exposure both at the low‐light exposure and light recovery phases. At the exposure phase, the low‐light treatments, especially when the light intensity is lower than the light compensation point, significantly decreased plant fresh weight and RGR, but increased plant height, stem internode distance, stem mass ratio, and stem epidermal cell length. The transcriptome information showed that the low light induced a reduction of photosynthetic capacity, which is in accord with the low fresh weight and RGR, while the up‐regulated pathways relating to the synthesis of phytohormones provided molecular basis for the increased plant height and internode distance. Moreover, the pathways relating to cell stress response such as DNA repair were enriched in the extremely low‐light treatment. At the light recovery phase, the plants from the low‐light treatments still had a low RGR, though the response of transcriptome showed an enhancement of energy metabolism. Moreover, the transcriptome information implies that the shift from low light to normal light exerted damage on the photosynthetic system and induced plant self‐protection. In addition, the enriched pathways relating to anthocyanin synthesis, following the shift from extremely low light to normal light, indicate an activation of photo‐protective mechanism. Our findings indicate the negative influences of low light on submerged macrophytes not only occur at the low‐light exposure phases but also after light recovery. Nevertheless, the macrophytes would adjust morphological traits to surrounding light environments. The transcriptome can show the detailed molecular basis of plant responses/acclimations, including but not limited to morphological performances. This study establishes a bridge connecting morphological and molecular responses/acclimations and contributes to aquatic ecosystem management and vegetation restoration.

## AUTHOR CONTRIBUTIONS

**Qingchun Guo:** Formal analysis (lead); investigation (lead); visualization (lead); writing – original draft (equal); writing – review and editing (equal). **Yuxuan Gao:** Formal analysis (supporting); writing – review and editing (supporting). **Chao Song:** Investigation (supporting). **Xinhou Zhang:** Conceptualization (equal); supervision (equal); writing – original draft (equal); writing – review and editing (lead). **Guoxiang Wang:** Conceptualization (equal); funding acquisition (lead); supervision (equal).

## CONFLICT OF INTEREST STATEMENT

The authors declare that there are no conflicts of interest.

## Data Availability

Transcriptomics data are publicly available from NCBI (BioProject ID PRJNA977960). Other data used in this study has been archived in Dryad Digital Repository https://doi.org/10.5061/dryad.2fqz612w1.

## 1

**TABLE A1:** Data filtering statistics.

Sample	Clean reads no.	Clean data (bp)	Clean reads %	Clean data %
CK1	44892884	6,778,825,484	93.99	93.99
CK2	40191138	6,068,861,838	94.03	94.03
CK3	41752620	6,068,861,838	93.98	93.98
ML1‐T1	43155888	6,516,539,088	93.79	93.79
ML2‐T1	48628976	7,342,975,376	94.35	94.35
ML3‐T1	39875896	6,021,260,296	93.82	93.82
EL1‐T1	42338468	6,021,260,296	94.00	94.00
EL2‐T1	46209208	6,977,590,408	94.48	94.48
EL3‐T1	44419664	6,707,369,264	93.94	93.94
ML1‐T2	40530556	6,120,113,956	92.81	92.81
ML2‐T2	39778786	6,006,596,686	92.81	92.81
ML3‐T2	47632102	7,192,447,402	93.40	93.40
EL1‐T2	45951180	6,938,628,180	93.60	93.60
EL2‐T2	39560860	6,938,628,180	93.09	93.09
EL3‐T2	37776000	5,704,176,000	93.07	93.07

*Note*: Sample: the sample name; Clean Reads No.: high quality sequence read number; Clean Data (bp): number of bases in high quality sequences; Clean Reads %: high quality sequence reads accounted for the percentage of sequencing reads; Clean Data %: the percentage of high quality sequence bases to sequencing; T1: low‐light exposure phase; T2: light recovery phase.

**TABLE A2:** Trinity assemble result analysis.

	Low light exposure phase		Light recovery phase	
	Transcript	Unigene	Transcript	Unigene
Total length (bp)	250,482,640	88,866,211	265,169,468	97,128,165
Sequence number	220293	101363	242048	117484
Max. length (bp)	16,706	16,706	15,750	15,750
Mena length (bp)	1137.04	876.71	1095.52	826.74
N50 (bp)	1766	1268	1717	1121
N50 Sequence No.	42707	18309	45758	21513
N90 (bp)	462	380	443	369
N90 Sequence No.	152786	75006	168996	88254
GC%	44.71	44.04	44.63	43.92

*Note*: Total Length (bp): total sequence length; Sequence Number: sequence total; Max. Length (bp): maximum sequence length; Mena Length: average sequence length; N50 (bp): Arrange all the sequences from the longest to the shortest, add the sequence lengths in that order, and when the sum reaches 50% of the total length of the sequence, the length of the last sequence; N50 (bp): Arrange all the sequences in order of length from longest to shortest, add the sequence lengths in that order, and when the length of the sum reaches 90% of the total length of the sequence, the length of the last sequence; N50 Sequence No.: The total number of sequences with length greater than N50; N90 Sequence No.: The total number of sequences with length greater than N90; GC%: GC content of the sequence.

**TABLE A3:** Annotation results summary.

Database	Low light exposure phase		Light recovery phase	
	Number	Percentage	Number	Percentage
NR	41027	40.48	49745	42.34
GO	25570	25.22	32605	27.75
KEGG	18303	18.06	25434	21.65
Pfam	25304	24.96	33670	28.66
eggNOG	41864	41.30	50165	42.70
Swissprot	32199	31.77	41657	35.46

*Note*: Database: type of database; Number: represents the number of successfully annotated unigenes in the database; Percentage (%): Represents the ratio of the number of unigenes successfully annotated in the database to the total number of unigenes; In all database: The number of unigenes annotated in all databases.

**TABLE A4:** Top 20 list of enriched KEGG pathway in ML and EL treatment in low‐light exposure phase.

Pathway ID	Pathway	Class	DEGs (up, down)	FDR
ML				
**KO00196**	**Photosynthesis‐antenna proteins**	**Metabolism**	**23 (16, 7)**	**9.4E−08**
**KO00195**	**Photosynthesis**	**Metabolism**	**20 (3, 17)**	**1.3E−05**
KO04141	Protein processing in endoplasmic reticulum	Genetic Information Processing	48 (34, 14)	3.8E−04
KO04144	Endocytosis	Cellular Processes	35 (29, 6)	3.0E−03
**KO00940**	**Phenylpropanoid biosynthesis**	**Metabolism**	**14 (10, 4)**	**9.2E−03**
**KO00710**	**Carbon fixation in photosynthetic organisms**	**Metabolism**	**21 (7, 14)**	**1.5E−02**
KO00591	Linoleic acid metabolism	Metabolism	5 (4,1)	2.5E−02
KO03040	Spliceosome	Genetic Information Processing	31 (28, 3)	4.5E−02
KO00906	Carotenoid biosynthesis	Metabolism	5 (1, 4)	6.7E−02
KO00500	Starch and sucrose metabolism	Metabolism	17 (6, 11)	6.9E−02
KO00941	Flavonoid biosynthesis	Metabolism	5 (0,5)	9.4E−02
KO00270	Cysteine and methionine metabolism	Metabolism	15 (9, 6)	1.2E−01
KO04075	Plant hormone signal transduction	Environmental Information Processing	15 (6, 9)	1.8E−01
KO00943	Isoflavonoid biosynthesis	Metabolism	1 (1, 0)	2.1E−01
KO00052	Galactose metabolism	Metabolism	8 (5,3)	2.8E−01
KO00010	Glycolysis/Gluconeogenesis	Metabolism	21 (9, 12)	2.8E−01
KO01040	Biosynthesis of unsaturated fatty acids	Metabolism	5 (3, 2)	3.7E−01
KO00730	Thiamine metabolism	Metabolism	3 (1, 2)	3.7E−01
KO04626	Plant‐pathogen interaction	Organismal Systems	24 (11, 13)	4.7E−01
KO00040	Pentose and glucuronate interconversions	Metabolism	6 (2, 4)	4.7E−01
EL				
**KO00196**	**Photosynthesis ‐ antenna proteins**	**Metabolism**	**24 (14, 10)**	**5.9E−07**
**KO00195**	**Photosynthesis**	**Metabolism**	**23 (14, 9)**	**3.3E−06**
**KO00710**	**Carbon fixation in photosynthetic organisms**	**Metabolism**	**26 (13, 13)**	**4.5E−03**
KO00040	Pentose and glucuronate interconversions	Metabolism	11 (2, 9)	4.0E−02
KO04712	Circadian rhythm‐plant	Organismal Systems	9 (1, 8)	4.4E−02
**KO00940**	**Phenylpropanoid biosynthesis**	**Metabolism**	**14 (4, 10)**	**4.4E−02**
KO00730	Thiamine metabolism	Metabolism	5 (2, 3)	6.8E−02
KO03030	DNA replication	Genetic Information Processing	10 (10, 0)	6.8E−02
KO00906	Carotenoid biosynthesis	Metabolism	5 (1, 4)	1.5E−01
KO00941	Flavonoid biosynthesis	Metabolism	5 (0, 5)	2.2E−01
KO04075	Plant hormone signal transduction	Environmental Information Processing	17 (6, 11)	2.5E−02
KO00943	Isoflavonoid biosynthesis	Metabolism	1 (0, 1)	3.1E−02
KO01040	Biosynthesis of unsaturated fatty acids	Metabolism	6 (3, 3)	3.5E−01
KO00908	Zeatin biosynthesis	Metabolism	3 (3, 0)	3.5E−01
KO00905	Brassinosteroid biosynthesis	Metabolism	3 (1, 2)	3.5E−01
KO04016	MAPK signaling pathway‐plant	Environmental Information Processing	16 (3, 13)	3.5E−01
KO00500	Starch and sucrose metabolism	Metabolism	16 (2, 14)	3.6E−01
KO00130	Ubiquinone and other terpenoid‐quinone biosynthesis	Metabolism	5 (4, 1)	4.2E−01
KO00360	Phenylalanine metabolism	Metabolism	5 (2, 3)	4.7E−01
KO00480	Glutathione metabolism	Metabolism	14 (5, 9)	5.1E−01

*Note*: KEGG pathway identifiers and name, FDR are given. Shared enriched KEGG pathway (FDR < 0.05) between ML and EL treatment contrasts are in bold.

**TABLE A5:** Top 20 list of enriched KEGG pathway in ML and EL treatment in light recovery phase.

Pathway ID	Pathway	Class	DEGs (up, Down)	FDR
ML				
**KO00196**	**Photosynthesis‐antenna proteins**	**Metabolism**	**22 (3, 19)**	**5.0E−08**
KO03010	Ribosome	Genetic Information Processing	97 (60, 37)	5.2E−06
KO00010	Glycolysis/Gluconeogenesis	Metabolism	31 (23, 8)	1.2E−05
**KO00195**	**Photosynthesis**	**Metabolism**	**16 (6, 10)**	**4.6E−05**
**KO00190**	**Oxidative phosphorylation**	**Metabolism**	**33 (14, 19)**	**3.6E−03**
KO04141	Protein processing in endoplasmic reticulum	Genetic Information Processing	38 (26, 12)	6.6E−03
KO00051	Fructose and mannose metabolism	Metabolism	12 (12, 0)	1.8E−02
KO00940	Phenylpropanoid biosynthesis	Metabolism	9 (6, 3)	1.6E−01
KO00710	Carbon fixation in photosynthetic organisms	Metabolism	15 (11, 4)	1.6E−01
KO00030	Pentose phosphate pathway	Metabolism	10 (10, 0)	3.3E−01
KO00524	Neomycin, kanamycin and gentamicin biosynthesis	Metabolism	2 (2, 0)	4.8E−01
KO00520	Amino sugar and nucleotide sugar metabolism	Metabolism	11 (9, 2)	6.9E−01
KO00052	Galactose metabolism	Metabolism	6 (5, 1)	8.6E−01
KO04626	Plant‐pathogen interaction	Organismal Systems	19 (11, 8)	8.6E−01
KO00270	Cysteine and methionine metabolism	Metabolism	10 (8, 2)	8.6E−01
KO00620	Pyruvate metabolism	Metabolism	10 (7, 3)	1.0
KO03018	RNA degradation	Genetic Information Processing	9 (7, 2)	1.0
KO00254	Aflatoxin biosynthesis	Metabolism	1 (1, 0)	1.0
KO04070	Phosphatidylinositol signaling system	Environmental Information Processing	6 (3, 3)	1.0
KO00500	Starch and sucrose metabolism	Metabolism	9 (6, 3)	1.0
EL				
**KO00196**	**Photosynthesis‐antenna proteins**	**Metabolism**	**35 (4, 31)**	**1.0E−14**
KO00941	Flavonoid biosynthesis	Metabolism	12 (9, 3)	6.2E−07
**KO00195**	**Photosynthesis**	**Metabolism**	**22 (8,14)**	**1.5E−06**
KO04712	Circadian rhythm‐plant	Organismal Systems	14 (5, 9)	2.1E−06
**KO00190**	**Oxidative phosphorylation**	**Metabolism**	**50 (16, 34)**	**1.9E−05**
KO00940	Phenylpropanoid biosynthesis	Metabolism	18 (13, 5)	1.8E−04
KO00942	Anthocyanin biosynthesis	Metabolism	3 (2, 1)	9.7E−03
KO00945	Stilbenoid, diarylheptanoid and gingerol biosynthesis	Metabolism	5 (4, 1)	1.1E−02
KO04016	MAPK signaling pathway‐plant	Environmental Information Processing	20 (12, 8)	3.5E−02
KO00010	Glycolysis/Gluconeogenesis	Metabolism	27 (14, 13)	6.0E−02
KO00360	Phenylalanine metabolism	Metabolism	7 (4, 3)	1.1E−01
KO04070	Phosphatidylinositol signaling system	Environmental Information Processing	12 (8, 4)	1.1E−01
KO00500	Starch and sucrose metabolism	Metabolism	17 (11, 6)	1.6E−01
KO00943	Isoflavonoid biosynthesis	Metabolism	1 (0, 1)	2.0E−01
KO04141	Protein processing in endoplasmic reticulum	Genetic Information Processing	41 (24, 17)	2.3E−01
KO00710	Carbon fixation in photosynthetic organisms	Metabolism	18 (13, 5)	2.6E−01
KO04626	Plant‐pathogen interaction	Organismal Systems	29 (14, 15)	2.6E−01
KO00944	Flavone and flavonol biosynthesis	Metabolism	1 (1, 0)	4.6E−01
KO00904	Diterpenoid biosynthesis	Metabolism	2 (2, 0)	4.6E−01
KO00460	Cyanoamino acid metabolism	Metabolism	6 (3, 3)	5.3E−01

*Note*: KEGG pathway identifiers and name, FDR are given. Shared enriched KEGG pathway (FDR <0.05) between ML and EL treatment contrasts are in bold.
